# Faecal Indicator Bacteria and *Pseudomonas aeruginosa* in Marine Coastal Waters: Is there a Relationship?

**DOI:** 10.3390/pathogens9010013

**Published:** 2019-12-21

**Authors:** Adriana P. Januário, Clélia N. Afonso, Susana Mendes, Maria J. Rodrigues

**Affiliations:** MARE – Marine and Environmental Sciences Centre, ESTM, Politécnico de Leiria, 2520-630 Peniche, Portugal; clelia@ipleiria.pt (C.N.A.); susana.mendes@ipleiria.pt (S.M.); maria.rodrigues@ipleiria.pt (M.J.R.)

**Keywords:** water quality, bathing waters, faecal contaminant indicators, *Pseudomonas aeruginosa*

## Abstract

To estimate the quality of coastal waters, European Union Directive 2006/7/EC provides guidelines to assess levels of faecal bacteria, including *Escherichia coli* and intestinal enterococci. These microbiological criteria are based on studies that determine the risk of bathers having diseases caused by enteric bacteria, not necessarily measuring the potential danger associated with the presence of nonenteric pathogens. The association between the presence of faecal contaminant indicators and nonenteric pathogenic microorganisms has not been well defined yet. The purpose of this study is to establish a relationship between *Pseudomonas aeruginosa* and microbiological indicators of faecal contamination. Presence of microbiological contamination in the coastal waters near the sewage treatment plant (STP) of Peniche (Portugal) was confirmed (*P. aeruginosa* 135.8 Colony Forming Unit/100 mL, *Escherichia coli* 1100.1 Most Probable Number/100 mL, intestinal enterococci 2685.9 MPN/100 mL) with much lower levels in the areas located south of the STP, along the main water coastal current (beach 1: 0.7 CFU/100 mL, 16.5 MPN/100 mL, 100.5 MPN/100 mL; beach 2: 0.3 CFU/100 mL, 74.0 MPN/100 mL, 145.9 MPN/100 mL, respectively). Analysis of Pearson’s correlation revealed a strong positive correlation between *E. coli* and *P. aeruginosa*, suggesting *E. coli* as an indicator of *P. aeruginosa* presence.

## 1. Introduction

Beaches and coastal areas provide pleasant recreational environments, leading to an increase in tourism demand that has stimulated the development of these areas [1]. Consequently, intensified anthropogenic pressures on beaches are affecting the environmental quality, recreational experience, and human well-being [2]. Pollution of coastal areas is now an increasing concern for public health organisations and the general public [3]. High quality standards must be met in these coastal waters, so that people can appreciate them without compromising their health [4,5].

The monitoring programs that are in place to assess the quality of bathing waters at the microbiological level are based on counts of bacterial indicators related to faecal contamination, such as *Escherichia coli* and intestinal enterococci [6]. Regular monitoring of bathing water is an absolute need in order to protect the health of bathers and ensure high water quality. Members of the European Union states apply the European Bathing Water Directive (Directive 2006/7/EC) [7] to monitor and characterize the quality of coastal bathing waters. This directive defines criteria for categorizing bathing water in four different levels—poor, sufficient, good, or excellent—based on microbial quality, by specifying the acceptable counts of *E. coli* and intestinal enterococci, which are monitored as microbiological parameters [8,9,10].

Although broadly used, traditional faecal indicators may be insufficient for determining the risk of disease from nonenteric pathogens involved in skin, eye, respiratory tract, nose, ear, and throat diseases [11]. These indicators were based on conclusions from epidemiological studies that implicate the enteric bacteria *E. coli* and enterococci in cases of gastroenteritis associated with swimming. However, the majority of recently reported cases of infectious diseases linked with coastal bathing waters are of nonenteric origin [12], creating a requirement for nonenteric indicator organisms that must be additionally evaluated. Opportunistic bacteria, such as *Pseudomonas aeruginosa* [13], might better serve to indicate the occurrence of some of the nonfaecal pathogens [12], especially in bathing waters [14]. Taking into account the results found in a study from Mariño and coworkers, where a positive correlation was found between skin infections and the presence of *P. aeruginosa* in seawater [14], this opportunistic bacteria [13] might better serve to indicate the occurrence of some nonfaecal pathogens in bathing waters [12].

Being rod-shaped, gram-negative, facultative aerobic bacterium, *P. aeruginosa* has minimal survival requirements [15,16] and a remarkable adaptation ability towards a variety of environmental conditions, being able to thrive in soil, water, animal hosts, hospital settings, soap, and even distilled water [17,18,19,20]. *P. aeruginosa*, as the origin of an extensive variety of infections, is a main cause of illness in immunocompromised individuals [21]. Some examples are endocarditis, urinary tract infections, pneumonia, gastrointestinal infections, and meningitis, and it is also a leading cause of septicaemia [19]. *P. aeruginosa* is the pathogen mostly implicated in folliculitis and ear infections (otitis externa) acquired by contact with recreational waters containing the bacterium [19,22,23].

The aims of this study are to detect and enumerate *P. aeruginosa* in the coastal waters of Peniche (Portugal), and to establish a relationship between this opportunistic pathogen and the faecal contamination indicators (intestinal enterococci and *E. coli*).

## 2. Results

Intestinal enterococci and *E. coli* were enumerated (MPN/100 mL), and *P. aeruginosa* samples were counted (CFU/100 mL) at three sampling sites (the sewage treatment plant (STP), beach 1, and beach 2), for 6 months (Table 1).

The higher loads of both faecal indicators and *P. aeruginosa* counts were obtained from the sewage treatment plant (STP) sampling site, where the water can be classified as having poor quality, and the values of microbiological indicators are higher when compared to the values established by law [1]. The same assessment can be made for the remaining two sites, beach 1 and beach 2, having exceeded the safety limits, although the difference between the criteria threshold and the actual value obtained was lower.

**Figure 1:**
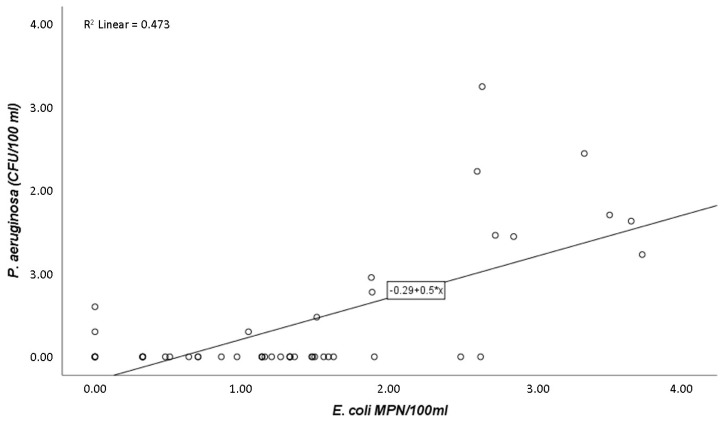
Scatter diagram indicating the relationship between *P. aeruginosa* (CFU/100 mL) and *E. coli* (MPN/100 mL) counts (r = 0.7; *p*-value ≤ 0.05).

**Figure 2:**
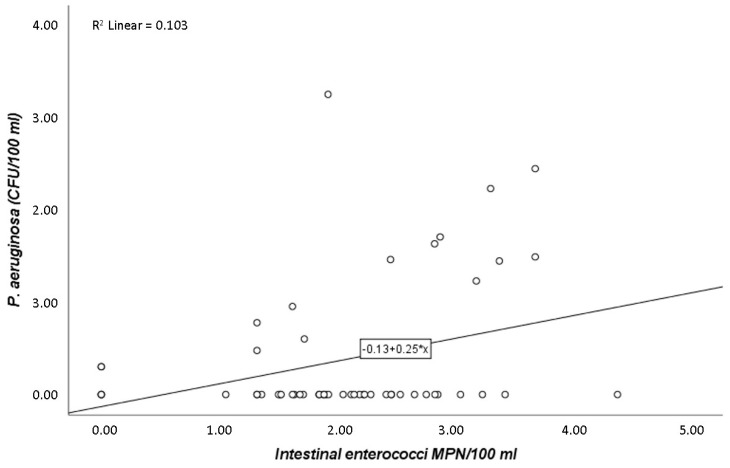
Scatter diagram indicating the relationship between *P. aeruginosa* (CFU/100 mL) and intestinal enterococci (MPN/100 mL) counts (r = 0.3; *p*-value ≤ 0.05).

Pearson’s correlation coefficient (r) showed that *P. aeruginosa*, when correlated only with *E. coli*, presented a strong positive association (r = 0.7; *p*-value ≤ 0.05). Thus, variations of *P. aeruginosa* are explained in 47.3% of cases by *E. coli* (Figure 1). Equally significant, but with a much less pronounced tendency, was the relationship described between *P. aeruginosa* and intestinal enterococci (r = 0.3; *p*-value ≤ 0.05) (Figure 2).

**Figure 3:**
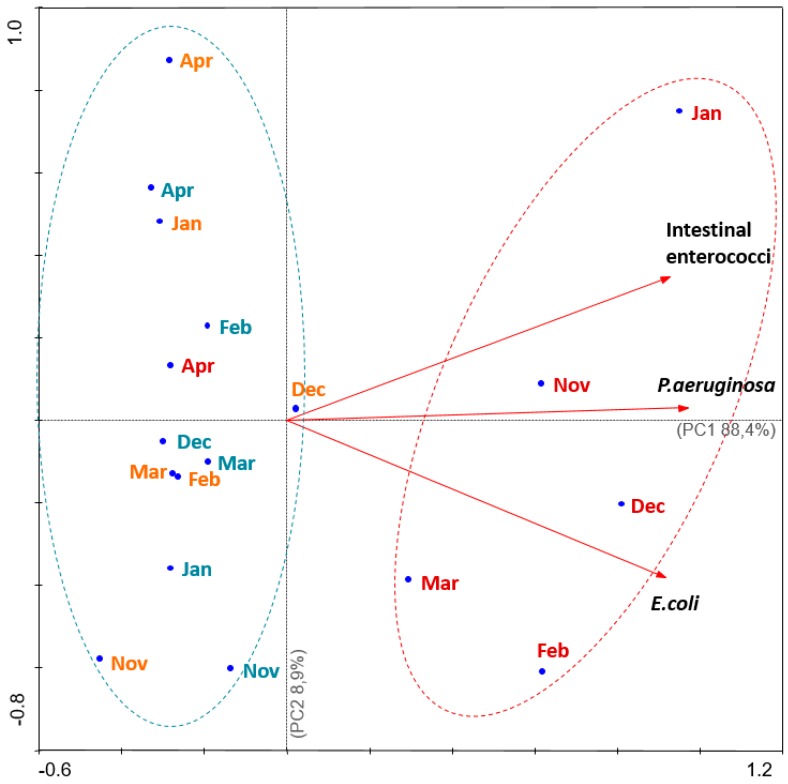
Principal component analysis (PCA) biplot of intestinal enterococci, *E. coli*, *P. aeruginosa*, and the sampling sites over the 6 months of the experiment. Note: months represented in red—sewage treatment plant (STP); months represented in blue—beach 1; months represented in orange—beach 2.

First and second principal components (PC1 and PC2, respectively) describe 97.3% of the original information and reinforce the differences between the characteristics of the sampling sites (Figure 3), with PC1 being the most significant component, expressing 88.4% of the total variance. In addition, the Principal Component Analysis biplot demonstrated a pattern of contamination characterized by STP in winter months (red group, Figure 3). On the other hand, there are beach 1 and beach 2, which show lower contamination patterns (Figure 3). This explains the positive and strong correlation between *P. aeruginosa* and the faecal bacteria indicators (intestinal enterococci and *E. coli*), clearly demonstrated by the position of the vectors, such as equal direction and the formation of an acute angle (Figure 3).

## 3. Discussion

Directive 2006/7/EC of the European Parliament and of the council from 15 February 2006, regarding the management of bathing water quality, provide clear guidelines to assess inland, coastal, and transitional water quality by the enumeration of microbiological parameters, such as intestinal enterococci and *E. coli*, indicating the potential presence of microbiological hazards [7]. In view of this, the main goal of the present study was to understand if there was a relationship between *Pseudomonas aeruginosa* and the faecal contamination indicators (intestinal enterococci and *E. coli*). Our results showed not only high numbers of intestinal enterococci and *E. coli*, but also the presence of *P. aeruginosa* in the water samples collected near the sewage treatment plant (STP) (Table 1, Figure 3). Previous studies reported high loads of *E. coli* and enterococci that exceeded the acceptable levels in the same coastal waters [24], and high numbers of bacteria were detected in limpets in this same spot [25]. In Europe, before coastline dischargement, sewage can receive secondary (organic matter removal) or tertiary (nutrients and bacteria removal) treatment. However, in about half of the European countries, most of the sewage treatment plants comprise only primary and secondary treatment [26], which can explain the high rate of faecal bacteria enumerated from the STP site (Figure 3). 

Because *E. coli* has an exclusively faecal origin, its occurrence in water endorses faecal contamination of the analysed water samples [10]. In this case, such contamination may originate from discharges from the STP (Figure 3). Literature shows that there is an epidemiological connection between gastrointestinal diseases among bathers and poor water quality [2], and sewage pollution of water bodies carries a risk to human health through waterborne pathogens [27]. Vergine et al. showed that the counting capability of Colilert-18 presented a significantly higher recovery of *E. coli* than the classical membrane filtration method when evaluating wastewater samples from different origins and levels of treatment. Thus, Colilert-18 is an effective method for enumerating *E. coli* at the STP site, which contains a mix of coastal waters and treated wastewaters [28]. Another study showed that Enterolert-E detected similar proportions of *Enterococcus* species in marine waters compared with a standard reference method (Environmental Protection Agency Method 1600) [29].

It is known that *P. aeruginosa* can be found at locations near the runoff of treatment plants [30], resulting in contaminated waters that are frequently described as a vehicle for infections [16,31,32]. In the present study, there were mean counts of 135.8 CFU/100 mL of *P. aeruginosa* in the water collected near the STP runoff. In one month, there was a maximum of 1800 CFU/100 mL, which is a very high and alarming number (Table 1, Figure 3). Lower counts had been reported before at locations upstream of sewage treatment plants, where the *P. aeruginosa* counts were 2–33 CFU/100 mL, while at a downstream site, the level was 350 CFU/100 mL in an emergency raw sewage overflow pipe [33]. 

The most relevant conclusion of this study is that *E. coli* counts are statistically significant in explaining the variations of *P. aeruginosa*. Intestinal enterococci and *E. coli* have been the only mandatory parameters for the assessment of the water quality at bathing sites [34], and because of this, it was expected that the numbers of these coliforms would correlate (being statistically significant) with the presence of the pathogen. Accordingly, our results revealed that *E. coli* numbers correlate with *P. aeruginosa* presence, despite the nonenteric nature of this pathogen. *E. coli* was earlier described as the best-known indicator of pathogenic microbes (although of faecal origin) in water [10], which is in accordance with the findings of the present study. Additionally, Pearson’s correlation between *P. aeruginosa* and *E. coli* is 0.687, and variations of *P. aeruginosa* are explained in 47.3% of cases by *E. coli* (Figure 1). This result points to a strong correlation, which leads to a positive and important relationship between them. When it comes to intestinal enterococci, this indicator parameter does not explain the numbers of *P. aeruginosa*, suggesting that intestinal enterococci may not be a good indicator of *P. aeruginosa* presence, since its correlation had a much less pronounced tendency (r = 0.3; *p*-value ≤ 0.05) (Figure 2). Curiously, previous studies have advised that introduction of intestinal enterococci as a quality parameter could cause higher failures of microbiological standards, leading to more cases of beach closures [35]. On the other hand, some authors suggest that intestinal enterococci may be better indicators of human health dangers in bathing coastal waters rather than *E. coli* [36], which is not in accordance with the present results.

The statistically significant relationships between *P. aeruginosa* and *E. coli* (Figure 1) and between *P. aeruginosa* and intestinal enterococci (Figure 2) are also represented in the PCA analysis (Figure 3). It is showed that the three locations under study can be separated into two major areas: one area was heavily contaminated (STP), where the counts of the microorganisms were very high; and a second area was less contaminated (beach 1 and beach 2), where the enumerations were lower. The information obtained from the PCA analysis (Figure 3) is consistent with the statistical significance of the relationship of the parameters in the study (*P. aeruginosa* and *E. coli*, Figure 1; *P. aeruginosa* and intestinal enterococci, Figure 2). This can still be explained by the discharged waters from the STP and their probably poorly disinfection. Corroborating this, Silva et al. [25] performed microbiological analysis of limpet samples near the same STP site and other surrounding areas. Their findings suggest that limpets near the STP discharges have higher levels of *E. coli* compared with the other two sites in the geographical area, with lower levels of the same bacteria.

Beach 1 and beach 2 are places of recreational activity that are visited by bathers. During the months under study, the quality of water was evaluated and considered poor (Table 1). However, this was not representative of the bathing season, where the presence of more bathers is expected, along with the increase of water temperatures. It is reported that elevated water temperatures in summer stimulate *P. aeruginosa* growth [32], which can explain the relatively low numbers of this pathogen during the set of months analysed, namely at the end of autumn, in winter, and in early spring (Beach 1: mean of 0.7 CFU/100 mL, maximum 8 CFU/100 mL; Beach 2: mean of 0.3 CFU/100 mL, maximum of 5 CFU/100 mL). The exposure conditions and the dermal route of infection by *P. aeruginosa* are not well-defined [23]; nonetheless, it is known that even lower concentrations of this pathogen can cause illness in both normal and immunocompromised humans [19]. *P. aeruginosa* in untreated recreational waters has been linked with otitis externa in bathers [37,38], as well as other health problems. For instance, otitis externa may occur when levels of *P. aeruginosa* exceed 11 CFU/100 mL, which was demonstrated in a study by Strathman and coworkers [30]. Two outbreaks of this disease were documented in three lakes, revealing the presence of *P. aeruginosa* in both water samples and patient ear swabs. The concentrations of the bacteria varied from 2–37 CFU/100 mL to 311–736 CFU/100 mL in the third lake [30]. Other study corroborating the same infection pattern of otitis externa also highlighted the amplified risk with the exposure period to the contaminated waters. In this case, 69% of the water samples had only 4 CFU/L of *P. aeruginosa* [39]. These cases confirm that even lower levels of *P. aeruginosa* in bathing waters may be associated with an enhanced risk of ear infections, particularly otitis externa. This leads to a reasonable concern in monitoring bathing waters near polluted sites, such as near sewage treatment plants, where high levels of faecal bacteria have been found, especially as a positive correlation has already been established in seawater between skin infections and the presence of *P. aeruginosa* [14].

Moreover, there is still a lack of information available regarding the correlation between faecal indicator bacteria and pathogenic microorganism concentrations [40]. Additionally, Zabed and colleagues [41] pointed out that the enumeration of these faecal contaminants may be insufficient to assess the risk of nonenteric pathogens, such as *Staphylococcus aureus* and *P. aeruginosa*, which have been detected in recreational waters.

In the present research, a positive correlation was observed between *E. coli* and *P. aeruginosa* numbers, so these results reinforce the information given by the official quality indicator parameters (for intestinal enterococci and *E. coli*). However, the authors believe it is advisable to add the enumeration of *P. aeruginosa* to the quality control of coastal waters, as this is a nonenteric pathogen, and the observed positive correlation might not always be true, especially in the summer, when the numbers of *P. aeruginosa* might reach even higher values than those observed in the present study. Additionally, the relationship between *E. coli* and *P. aeruginosa* numbers should be studied in colder coastal waters, such as northern countries. Because *E. coli* is mesophilic and *P. aeruginosa* is psychrophilic, it is possible that the survival time of *E. coli* might be reduced compared to the survival time of *P. aeruginosa* in the natural environment.

## 4. Materials and Methods 

### 4.1. Study Area and Sample Collection

**Figure 4:**
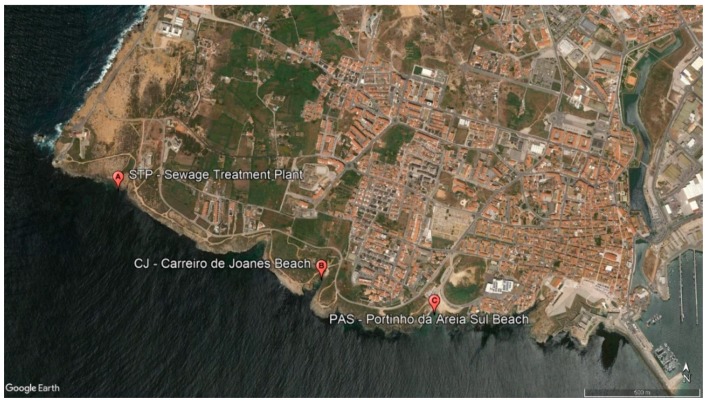
South coast of Peniche (Portugal), showing the sampling sites: (**A**) sewage treatment plant (STP; 39.358447°, −9.405446°), (**B**) beach 1 (CJ; 39.354758°, −9.394529°), and (**C**) beach 2 (PAS; 39.353452°, −9388739°). Image generated from Google Earth Pro software.

Water samples (1 litre) were collected during low tide, approximately 30 cm below the water level, at three sampling sites in Peniche south coast (Portugal) for six consecutive months: November, December, January, February, March, and April. The first site was chosen because of its close proximity with the sewage treatment plant runoff (STP; 39.358447°, −9.405446°). To understand if there is contamination and this is scattered through the currents along the south coast, two more spots were chosen: beach 1—Carreiro de Joanes beach (CJ; 39.354758°, −9.394529°); and beach 2—Portinho da Areia Sul beach (PAS; 39.353452°, −9388739°), approximately 1.03 km and 1.55 km southeast from STP, respectively (Figure 4). A total of 54 samples (Table 1) were duly packed in thermal bags and transported to the laboratory. Samples were analysed within 6 hours of completion.

### 4.2. Enumeration of E. coli and Intestinal Enterococci

The Colilert-18 (ISO 9308-2:2012, IDEXX Laboratories, Inc., Westbrook, ME, USA) and Enterolert-E (ISO 7899-1, IDEXX Laboratories, Inc., Westbrook, USA) MPN method procedures were executed according to the company instructions (IDEXX Laboratories, Inc., Westbrook, ME, USA) for the enumeration of *Escherichia coli* and intestinal enterococci, respectively. Three dilutions were performed (1:10; 50:50; 9:10) due to the suspicion of the high microbiological load. Briefly, 100 mL of each diluted sample and one vial of Colilert-18 and Enterolert-E were added to sterile vessels, capped and shaken to dissolve the reagent, the content was transferred to a 97-well Quanti-Tray (IDEXX Laboratories, Inc., Westbrook, USA), heat-sealed in the Quanti-Tray Sealer (IDEXX Laboratories, Inc., Westbrook, ME, USA), and incubated at 35 ± 0.5 °C for 18 hours (Colilert-18) or 41 ± 0.5 °C for 24 hours (Enterolert-E). After incubation, Quanti-Trays were observed under ultra-violet (UV) light (365 nm) and the fluorescent cells were counted and referred to the MPN table provided by IDEXX for the enumeration of *E. coli* and intestinal enterococci. Microbiological counts were reported as MPN/100 mL.

### 4.3. Enumeration of Pseudomonas aeruginosa

For the detection and enumeration of *P. aeruginosa* in coastal water samples, the procedure described in ISO 16266:2006 was followed [42]. Briefly, 100 mL of each sample (no dilution, 1:10, 1:100) were filtered through a membrane filter of 0.45 μm and incubated on Cetrimide Agar (Biokar, France) plates, supplemented with 1% of glycerol (Himedia, India), at 36 ± 2 °C for 44 ± 4 h. Fluorescent colonies were considered confirmed for *P. aeruginosa* and reported as CFU/100 mL.

### 4.4. Statistical Analysis

Prior to statistical analysis, and to minimize the dominant effect of exceptional counts, enumerations of intestinal enterococci, *E. coli*, and *P. aeruginosa* were changed to log (x + 1) [43]. To evaluate the strength of the correlations between *P. aeruginosa* and intestinal enterococci, and between *P. aeruginosa* and *E. coli*, Pearson’s correlation coefficients (r) were determined [44]. All calculations were performed with IBM® SPSS® Statistics version 26 software (Copyright IBM Corp. © 1989–2019, Armonk, New York 10504-1722, USA). Results were considered significant at *p*-value ≤ 0.05. In addition, in order to identify patterns in the relation between intestinal enterococci, *E. coli*, and *P. aeruginosa* and the sampling sites, principal component analysis (PCA) was performed with CANOCO version 4.5 package (Copyright Petr Smilauer © 2012–2019, Ithaca, New York 14850, USA) [45]. This multivariate technique was used to identify the components (that is, the core variables) that explain the correlations within the measured data. Therefore, by means of PCA, it was possible to achieve associations between variables, reducing the dimension of the original data. The information provided by the principal components highlight the most meaningful parameters, describing the whole data matrix and affording data reduction with minimum loss of original information. Through a linear combination analysis, the positions of original variables in the diagram represent their relevantly interrelations. Thus, principal components effectively represent the original measured data. As a result, if the variables are closely positioned, their correlation is strong and positive. In contrast, if the variables are in an opposite position, then those variables are negatively correlated. Hence, graphical representation of PCA, which plots simultaneously the objects and the variables, is very useful to detect possible associations between variables and objects. Moreover, the association between objects and variables can be determined depending on their relationship and proximity within each group. In this study, PCA was executed to assess distribution patterns based on the parameters under study (*P. aeruginosa, E. coli*, intestinal enterococci) and the month-spot sampling. Although the results for the first two components (PC1 and PC2) were presented, the others were also analysed.

## Figures and Tables

**Table 1:** Means, sample size (n), minimum, and maximum counts for *P. aeruginosa*, *E. coli*, and intestinal enterococci from the three sampling sites: the sewage treatment plant (STP), beach 1 (Carreiro de Joanes, CJ), and beach 2 (Portinho da Areia Sul beach, PAS).

	STP				CJ (Beach 1)				PAS (Beach 2)			
	Mean	n	Minimum	Maximum	Mean	n	Minimum	Maximum	Mean	n	Minimum	Maximum
***P. aeruginosa*** **(CFU/100 mL)**	135.8	18	0.0	1800.0	0.7	18	0.0	8.0	0.3	18	0.0	5.0
***E. coli*** **(MPN/100 mL)**	1100.1	16	0.0	5172.0	16.5	18	0.0	74.0	14.1	18	0.0	75.0
**Intestinal enterococci** **(MPN/100 mL)**	2685.9	18	0.0	24196.0	100.5	18	0.0	455.8	145.9	18	0.0	720.2
